# Supplementary material for: Societal decisions about climate mitigation will have dramatic impacts on eutrophication in the 21st century
Source: Nat Commun. 2019 Feb 26;10:939. doi: 10.1038/s41467-019-08884-w (PMC6391408; doi:10.1038/s41467-019-08884-w)
Supplement: Supplementary file 1 — Supplementary Information [file 41467_2019_8884_MOESM1_ESM.pdf]

# Supplementary Information for

## Societal decisions about climate mitigation will have dramatic impacts on eutrophication in the 21$^{st}$ century

Sinha *et al.*

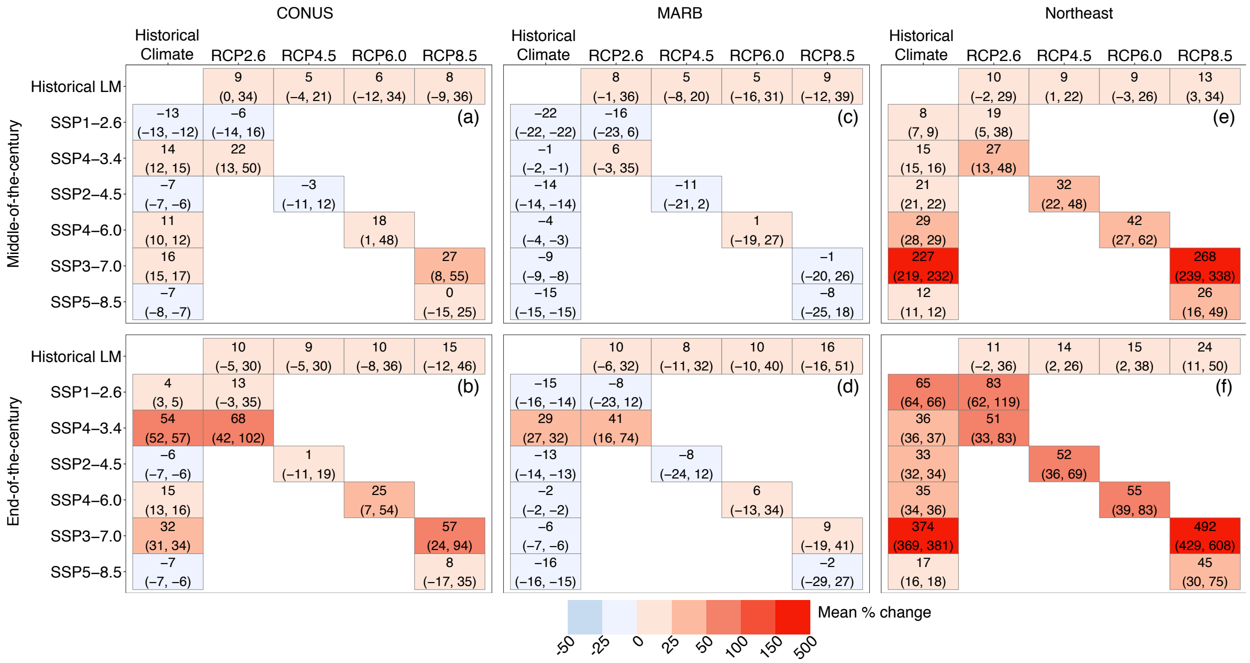


**Supplementary Figure 1:** Percentage change in mean total nitrogen flux by the middle-of-the-century (2031-2060) (top row) and end-of-the-century (2071-2100) (bottom row) periods relative to the historical period (1976-2005) for the SSP1-2.6, SSP4-3.4, SSP2-4.5, SSP4-6.0, SSP3-7.0, and SSP5-8.5 scenarios based on projected land use and fertilizer application rates, both with and without concomitant changes to climate. The effects of changes to climate in the absence of changes to land use and land management are denoted as the Historical LM scenario. Precipitation changes are based on RCP2.6 for SSP1-2.6 and SSP4-3.4, RCP4.5 for SSP2-4.5, RCP6.0 for SSP4-6.0, and RCP8.5 for SSP3-7.0 and SSP5-8.5. The projected changes are shown for the continental United States (CONUS), the Mississippi Atchafalaya River Basin (MARB), and the Northeast, with regions as outlined in Figure 1. The numbers in the boxes and their colors denote the mean percentage change across the CMIP5 models, while the numbers in parentheses represent the range across the CMIP5 models. Historical net anthropogenic nitrogen input was estimated based on the observational record, while future input was estimated using data from the LUH2 dataset.

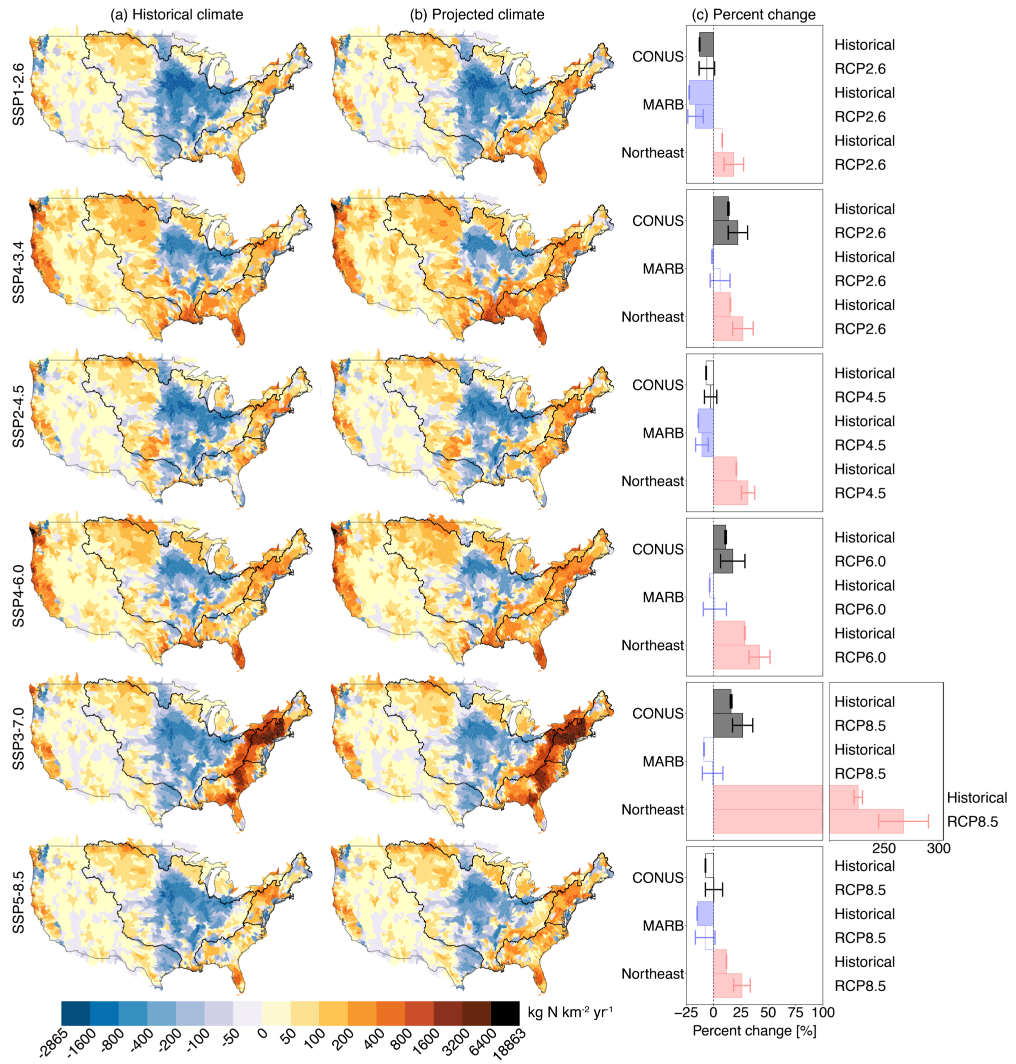


**Supplementary Figure 2.** Similar to Figure 2 but showing change in mean total nitrogen flux for the middle-of-the-century (2031-2060) relative to the historical period (1976-2005).

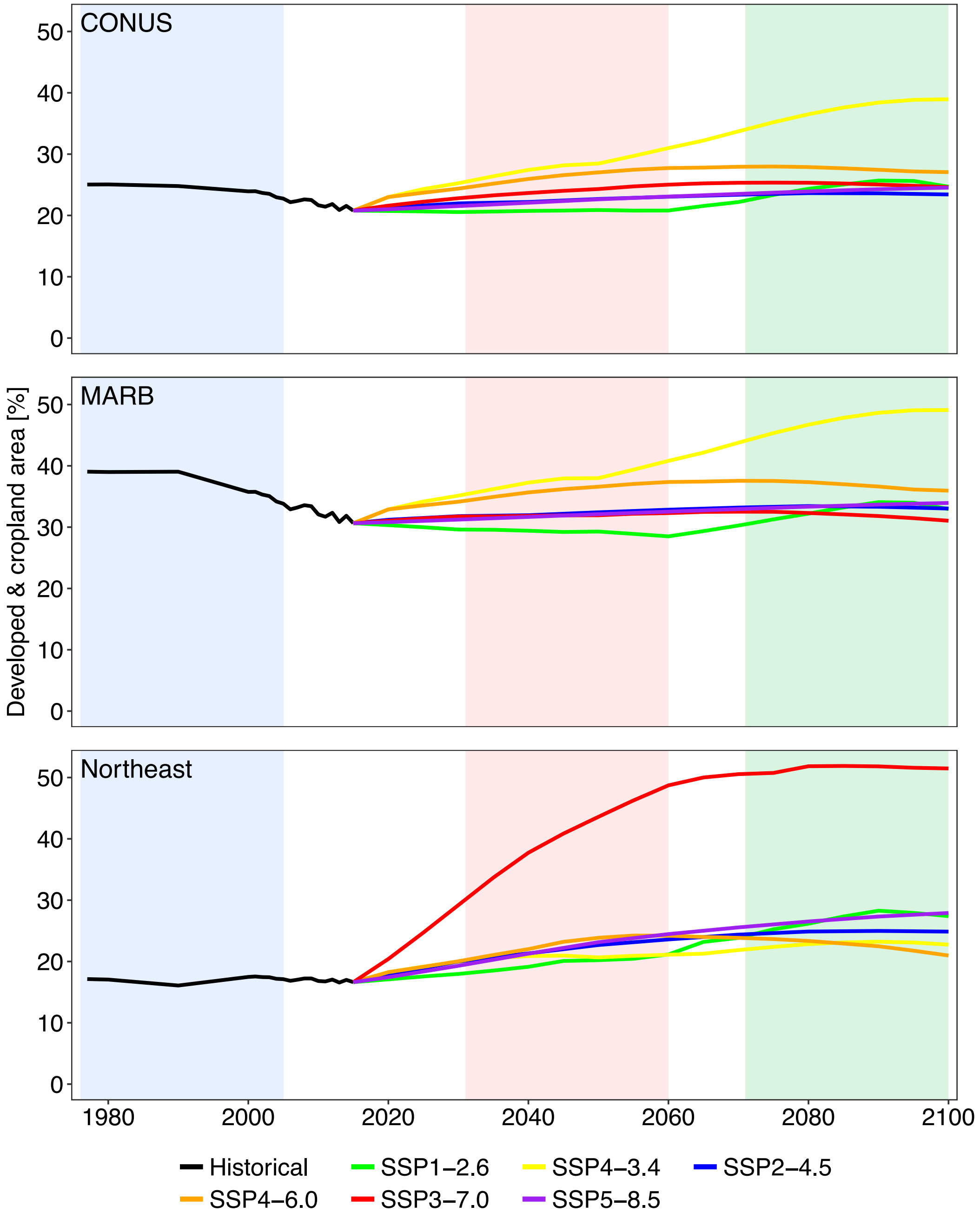


**Supplementary Figure 3.** Time series of percentage of land classified as developed and cropland based on the LUH2 database for the historical (light blue background), middle-of-the-century (light red background), and end-of-the-century (light green background) time periods for the six scenarios. The time series are shown for the continental United States (CONUS), the Mississippi Atchafalaya River Basin (MARB), and the Northeast, with regions as outlined in Figure 1.

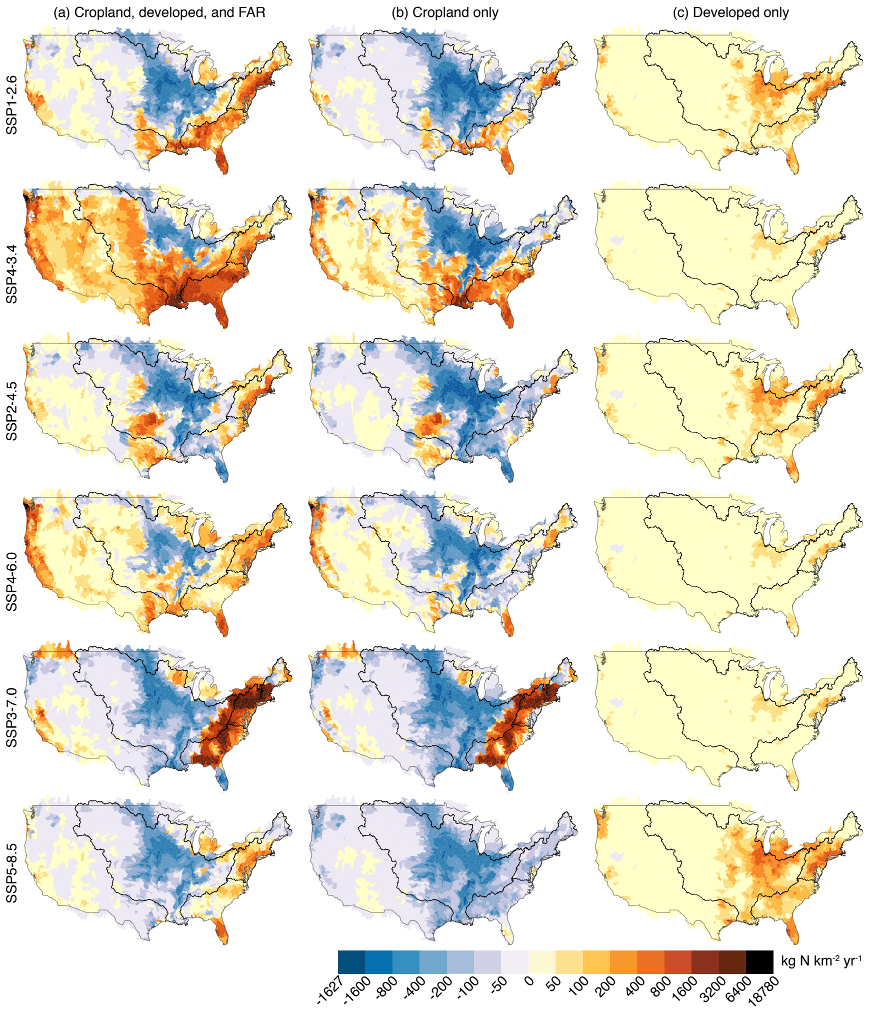


**Supplementary Figure 4.** Change in mean total nitrogen flux by the end-of-the-century (2071-2100) relative to the historical period (1976-2005) for the six scenarios based on (a) projected cropland, developed land, and fertilizer application rates, (b) projected cropland but historical developed land and fertilizer application rates, (c) projected developed land but historical cropland and fertilizer application rates, and (d) projected fertilizer application rates but historical land use. Precipitation is kept at historical levels. The black outline highlights the Mississippi Atchafalaya River Basin (MARB) and the Northeast regions. Net anthropogenic nitrogen input was estimated based on the LUH2 database for both the historical period and future period.

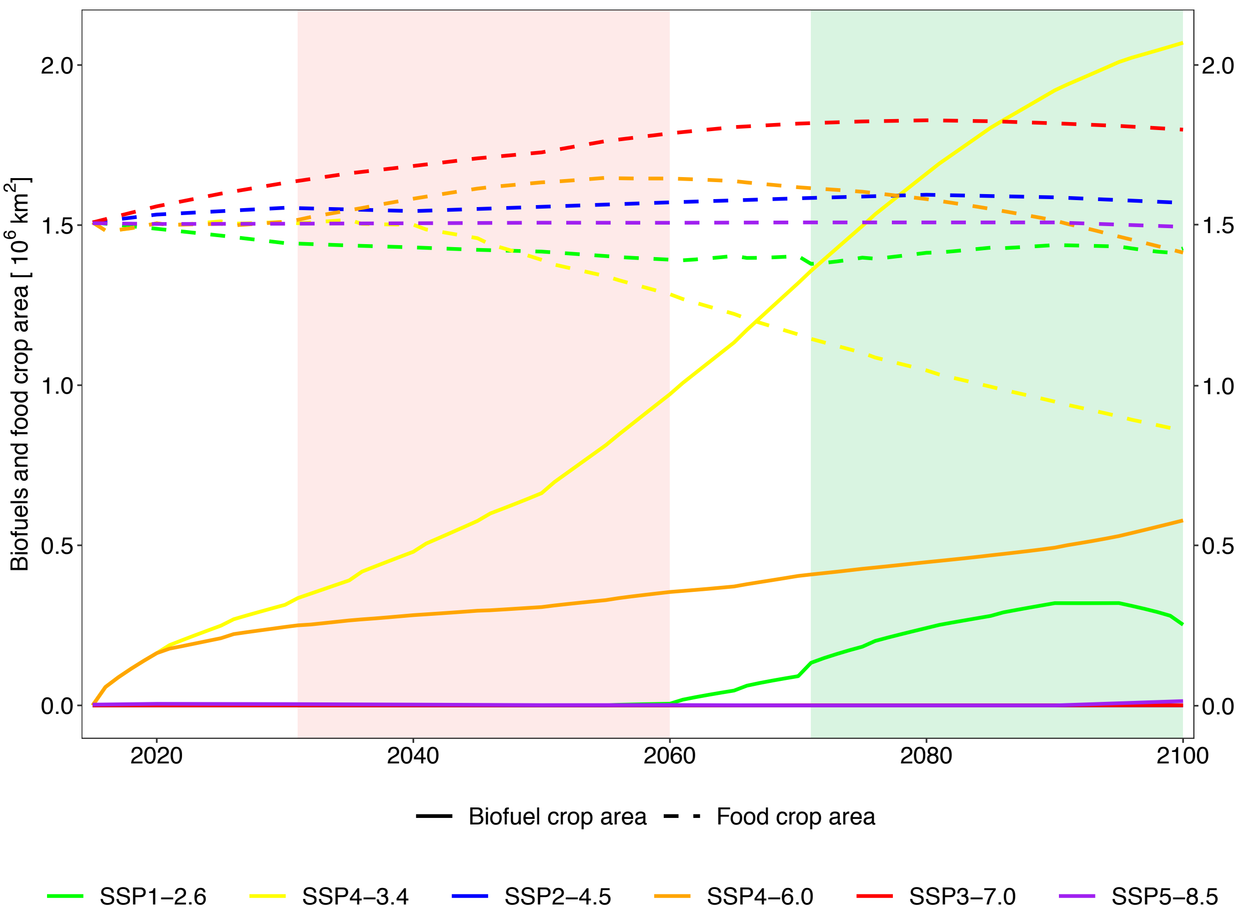


**Supplementary Figure 5.** Time series of land area used for 2nd generation biofuels and food crops based on the LUH2 database for the continental United States for the middle-of-the-century (light red background) and end-of-the-century (light green background) time period for the six scenarios examined here.

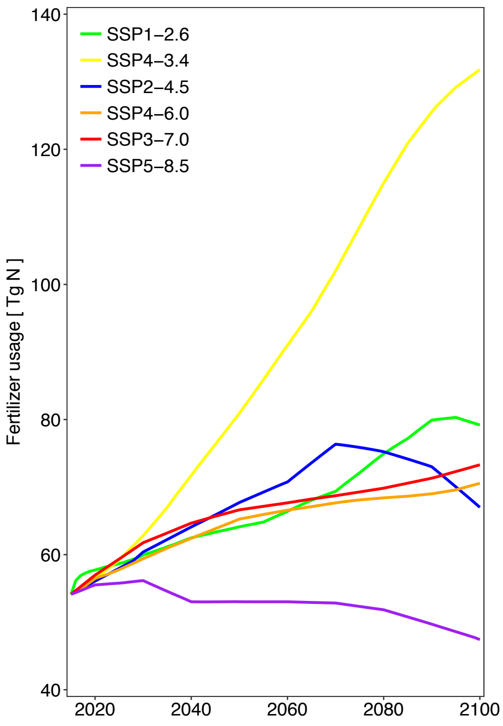


**Supplementary Figure 6.** Time series of projected fertilizer usage for countries falling in South, East, and Southeast Asia (see outline in Figure 5) for the six scenarios considered here. The primary countries in this region by area and population are China (47%, 35%), India (16%, 33%), and Indonesia (9%, 6%), where the numbers indicate the percent of the region's area and population in each country, respectively.

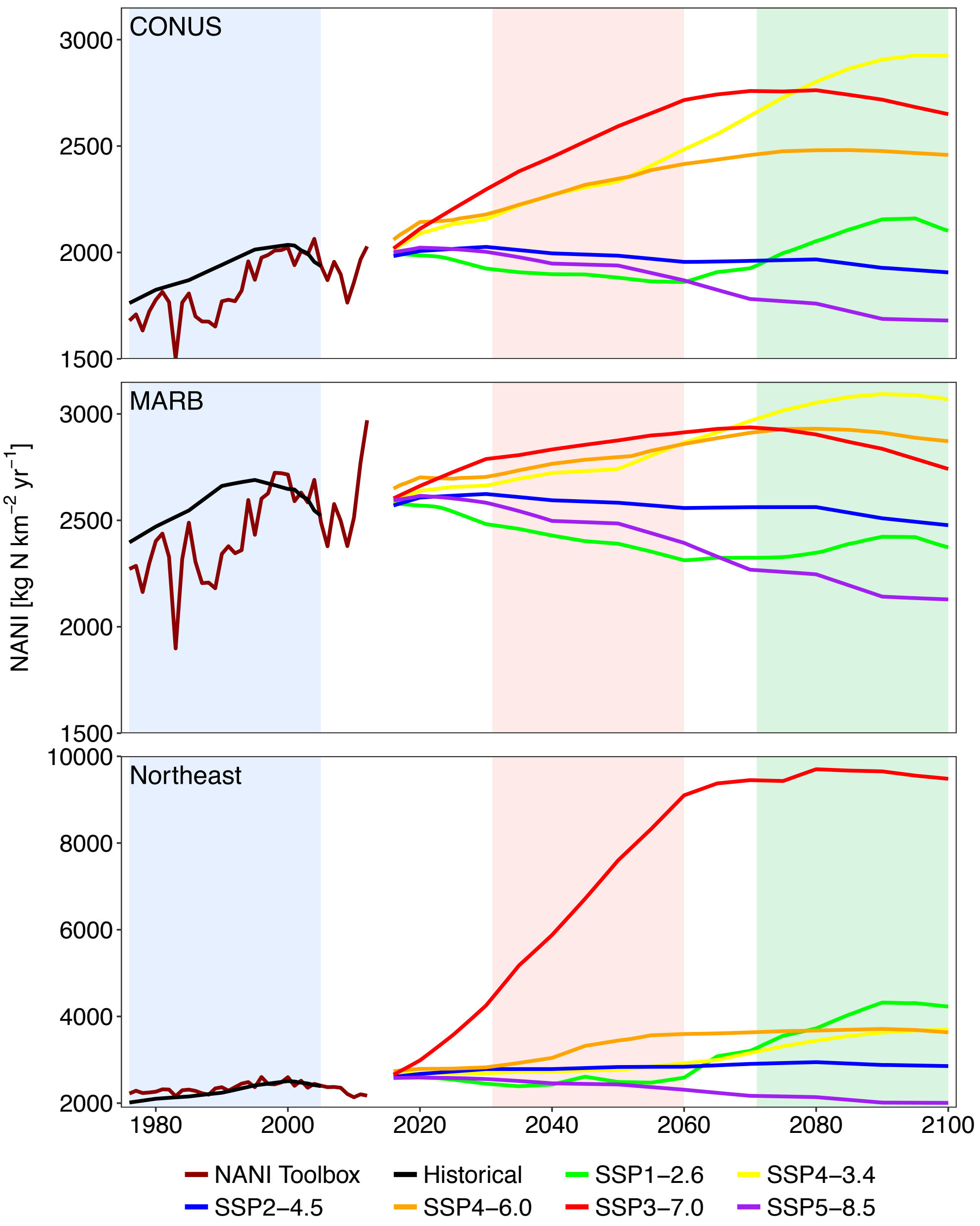


**Supplementary Figure 7.** Time series of NANI for the historical (light blue background) period based on the NANI toolbox (brown) and the LUH2 database (black), as well as for the middle-of-the-century (light red background) and end-of-the-century (light green background) time periods for the six scenarios considered here. Time series are shown for the continental United States (CONUS), the Mississippi Atchafalaya River Basin (MARB), and the Northeast, with regions as outlined in Figure 1.

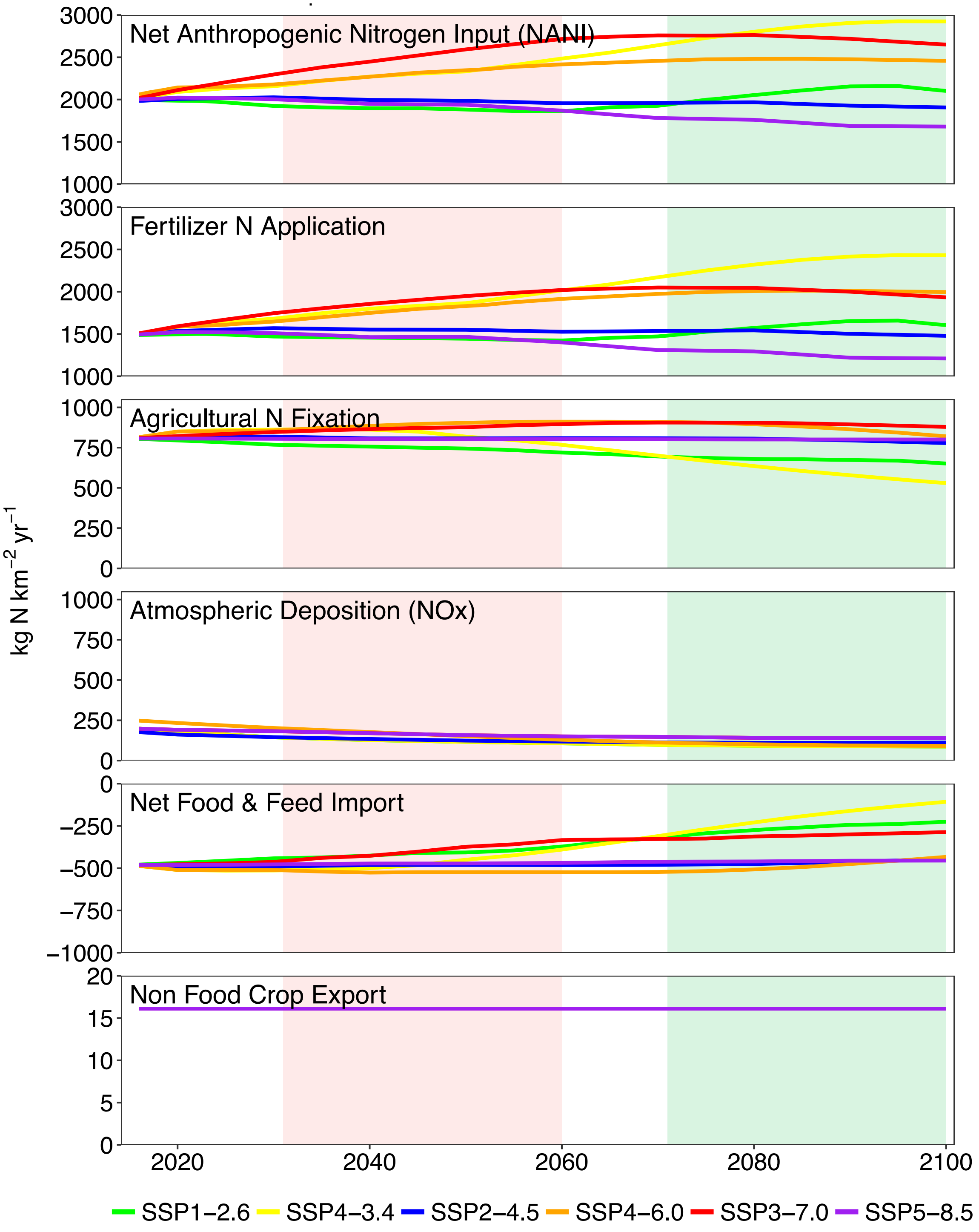


**Supplementary Figure 8.** Time series of total NANI and its components for continental United States for the middle-of-the-century (light red background) and end-of-the-century (light green background) time period for the six scenarios examined here.

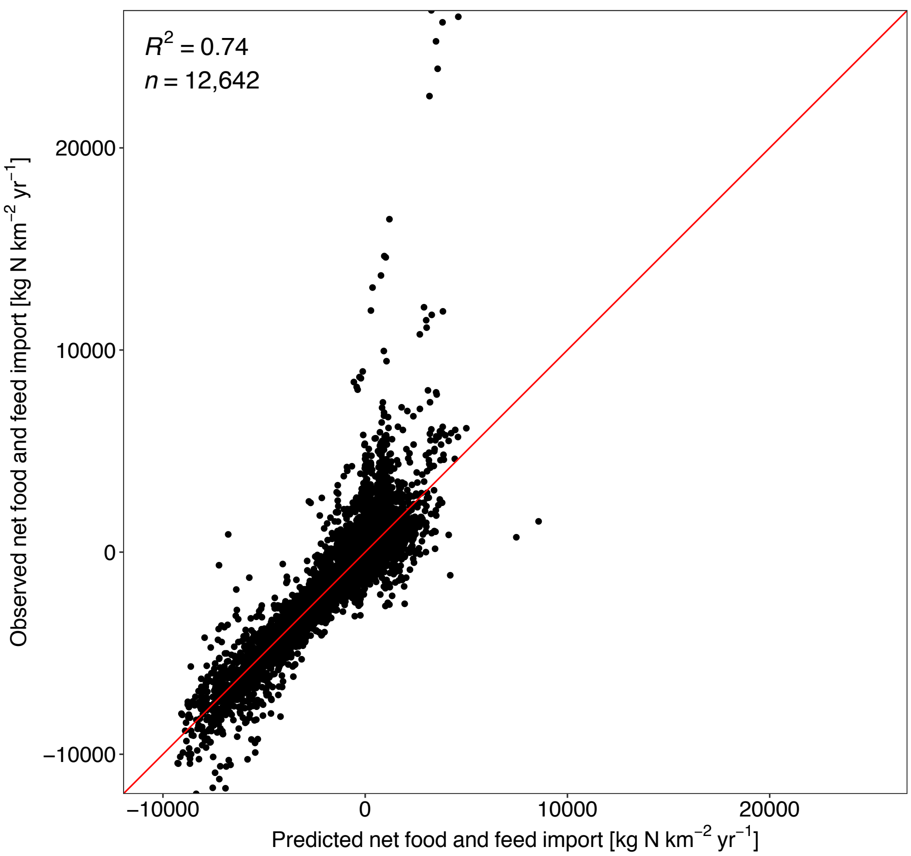


**Supplementary Figure 9.** Relationship between observed net food and feed import and predicted net food and feed import based on multilinear relationship with fertilizer application rate and agricultural nitrogen fixation rate for all HUC8 watersheds and for the six agricultural census years (1987, 1992, 1997, 2002, 2007, and 2012). The regressions were conducted separately by HUC2 region to capture regional differences in the relationship between net food and feed import, fertilizer application rates, and agricultural nitrogen fixation.

**Supplementary Table 1:** Fertilizer usage [Tg N/year] projected for 2015 and 2100 for the six LUH2 marker scenarios and for the GCAM IAM implementation of these six scenarios. The five different IAMs used in the LUH2 database are listed for scenarios that they contributed. In the LUH2 database, the SSP4-3.4 and SSP4-6.0 scenarios are contributed by the GCAM model, and the small difference between LUH2 and GCAM estimates for these scenarios is due to the harmonization process; these differences are not listed and are instead marked with a star. Differences between GCAM and LUH2 scenarios are only evaluated for the four scenarios for which GCAM did not contribute the marker scenario.

| | LUH2 | | | GCAM | | Difference |
|---|---|---|---|---|---|---|
| | Model | 2015 | 2100 | 2015 | 2100 | 2100 |
| SSP1-2.6 | IMAGE | 14.6 | 15.8 | 10.9 | 24.5 | 8.7 |
| SSP4-3.4 | GCAM | 14.6 | 24.7 | 11.5 | 25.4 | * |
| SSP2-4.5 | MESSAGE-GLOBIOM | 14.6 | 15.0 | 11.0 | 30.7 | 15.7 |
| SSP4-6.0 | GCAM | 14.6 | 19.8 | 11.5 | 20.7 | * |
| SSP3-7.0 | AIM | 14.6 | 18.9 | 11.0 | 22.8 | 3.90 |
| SSP5-8.5 | REMIND-MAGPIE | 14.6 | 11.9 | 11.0 | 16.2 | 4.3 |
| Range across SSP1-2.6, SSP2-4.5, SSP3-7.0, SSP5-8.5: | | | 7.0 | Average difference between models: | | 8.1 |

**Supplementary Table 2:** LUH2 land use categories and their combinations considered for model development

| Consolidated land use categories considered in model development | Land use categories included in LUH2 dataset |
|---|---|
| Forested land ($LU_F$) | forested primary land (primf) |
| | potentially forested secondary land (secdf) |
| Non-forested land ($LU_{NF}$) | non-forested primary land (primn) |
| | potentially non-forested secondary land (secdn) |
| Rangeland ($LU_R$) | Rangeland (range) |
| Developed land ($LU_D$) | urban land (urban) |
| Pasture ($LU_P$) | managed pasture (pastr) |
| Cropland ($LU_C$) | C3 annual crops (c3ann) |
| | C4 annual crops (c4ann) |
| | C3 perennial crops (c3per) |
| | C4 perennial crops (c4per) |
| | C3 nitrogen-fixing crops (c3nfx) |

**Supplementary Table 3.** Datasets used for continental United States and global analyses

| Dataset | Description | Source | Availability |
|---|---|---|---|
| BCCAv2-CMIP5 Climate daily Projections | CMIP5 precipitation projections at 1/8° for continental US | Downscaled CMIP3 and CMIP5 Climate and Hydrology Projections (DCHP) | http://gdo-dcp.ucllnl.org/downscaled_cmip_projections/dcpInterface.html |
| Future and historical land-use forcing dataset (LUH2 v2h & LUH2 v2f) | Future and historical land use forcing dataset that contains land use and global nitrogen fertilizer application rates | Land Use Harmonization (LUH2) | http://luh.umd.edu/data.shtml |
| Watershed Boundary Dataset (WBD) | Watershed boundaries for various hydrologic units within US | U. S. Geological Survey (USGS) | https://nhd.usgs.gov/wbd.html |
| Net Anthropogenic Nitrogen Inputs (NANI) Toolbox Version 3.1 | Net anthropogenic nitrogen input, including agricultural nitrogen fixation, net food and feed import, and non-food crop export components. Fertilizer and deposition components substituted as listed below. | Cornell University | http://www.eeb.cornell.edu/biogeo/nanc/nani/nani.htm |
| County-level estimates of nitrogen and phosphorus from commercial fertilizer for the Conterminous United States | Fertilizer application data for continental United States | U. S. Geological Survey (USGS) | https://doi.org/10.5066/F7H41PKX http://pubs.er.usgs.gov/publication/wri944176 http://pubs.er.usgs.gov/publication/ofr90130 |
| Precipitation-weighted mean annual atmospheric nitrate deposition | Atmospheric nitrogen wet deposition data | National Atmospheric Deposition Program (NADP) | http://nadp.sws.uiuc.edu/data/ntn/ |
| NOx deposition | Future NOx deposition at 1.9°×2.5° for the globe. | Jean-François Lamarque at the National Center for Atmospheric Research (NCAR) | https://acomstaff.acom.ucar.edu |

**Supplementary Table 4:** CMIP5 models used in this study

| Modeling Center (or Group) | Institute ID | Model Name |
|---|---|---|
| Commonwealth Scientific and Industrial Research Organization (CSIRO) and Bureau of Meteorology (BOM), Australia | CSIRO-BOM | ACCESS1.0 |
| Beijing Climate Center, China Meteorological Administration | BCC | BCC-CSM1.1 |
| College of Global Change and Earth System Science, Beijing Normal University | GCESS | BNU-ESM |
| Canadian Centre for Climate Modelling and Analysis | CCCMA | CanESM2 |
| National Center for Atmospheric Research | NCAR | CCSM4 |
| Community Earth System Model Contributors | NSF-DOE-NCAR | CESM1(BGC) |
| Centre National de Recherches Météorologiques / Centre Européen de Recherche et Formation Avancée en Calcul Scientifique | CNRM-CERFACS | CNRM-CM5 |
| Commonwealth Scientific and Industrial Research Organization in collaboration with Queensland Climate Change Centre of Excellence | CSIRO-QCCCE | CSIRO-Mk3.6.0 |
| NOAA Geophysical Fluid Dynamics Laboratory | NOAA GFDL | GFDL-CM3 |
| | | GFDL-ESM2G |
| | | GFDL-ESM2M |
| Institute for Numerical Mathematics | INM | INM-CM4 |
| Institut Pierre-Simon Laplace | IPSL | IPSL-CM5A-LR |
| | | IPSL-CM5A-MR |
| Japan Agency for Marine-Earth Science and Technology, Atmosphere and Ocean Research Institute (The University of Tokyo), and National Institute for Environmental Studies | MIROC | MIROC-ESM |
| | | MIROC-ESM-CHEM |
| Atmosphere and Ocean Research Institute (The University of Tokyo), National Institute for Environmental Studies, and Japan Agency for Marine-Earth Science and Technology | MIROC | MIROC5 |
| Max-Planck-Institut für Meteorologie (Max Planck Institute for Meteorology) | MPI-M | MPI-ESM-MR |
| | | MPI-ESM-LR |
| Meteorological Research Institute | MRI | MRI-CGCM3 |
| Norwegian Climate Centre | NCC | NorESM1-M |
